# Relation between local food environments and obesity among adults

**DOI:** 10.1186/1471-2458-9-192

**Published:** 2009-06-18

**Authors:** John C Spence, Nicoleta Cutumisu, Joy Edwards, Kim D Raine, Karen Smoyer-Tomic

**Affiliations:** 1 Faculty of Physical Education and Recreation, University of Alberta, Edmonton, Canada; 2 Capital Health, Edmonton, Canada; 3 Centre for Health Promotion Studies, School of Public Health, University of Alberta, Edmonton, Canada; 4 Department of Geography, University of Delaware, Newark, USA

## Abstract

**Background:**

Outside of the United States, evidence for associations between exposure to fast-food establishments and risk for obesity among adults is limited and equivocal. The purposes of this study were to investigate whether the relative availability of different types of food retailers around people's homes was associated with obesity among adults in Edmonton, Canada, and if this association varied as a function of distance between food locations and people's homes.

**Methods:**

Data from a population health survey of 2900 adults (18 years or older) conducted in 2002 was linked with geographic measures of access to food retailers. Based upon a ratio of the number of fast-food restaurants and convenience stores to supermarkets and specialty food stores, a Retail Food Environment Index (RFEI) was calculated for 800 m and 1600 m buffers around people's homes. In a series of logistic regressions, associations between the RFEI and the level of obesity among adults were examined.

**Results:**

The median RFEI for adults in Edmonton was 4.00 within an 800 m buffer around their residence and 6.46 within a 1600 m buffer around their residence. Approximately 14% of the respondents were classified as being obese. The odds of a resident being obese were significantly lower (OR = 0.75, 95%CI 0.59 – 0.95) if they lived in an area with the lowest RFEI (below 3.0) in comparison to the highest RFEI (5.0 and above). These associations existed regardless of the covariates included in the model. No significant associations were observed between RFEI within a 1600 m buffer of the home and obesity.

**Conclusion:**

The lower the ratio of fast-food restaurants and convenience stores to grocery stores and produce vendors near people's homes, the lower the odds of being obese. Thus the proximity of the obesogenic environment to individuals appears to be an important factor in their risk for obesity.

## Background

Food eaten away from home, especially fast-food, is associated with weight gain and the obesity epidemic [1-4]. Greater availability of fast-food restaurants has led to increased consumption of fast-food [5]. Furthermore, if supermarkets are not readily available then fast-food restaurants often serve as a substitute for food access [6]. Also, adolescents are obtaining less of their energy intake at home and more at restaurants and fast food places [7]. This behaviour is reasonable considering that fast-food is cheaper and more energy-dense per measure of weight than other healthier foods such as fruits and vegetables that are purchased in a grocery store [8]. In fact, the prices of fast-food and fruit and vegetables are differentially associated with dietary quality and adiposity among people living in the United States [9]. Thus, the location of fast-food restaurants in urban areas may contribute to the obesogenic environment [10].

A recent study in California found that the relative availability of different types of food retailers around individuals' homes was associated with the bodyweight status of residents [11]. Specifically, the Retail Food Environment Index (RFEI) was calculated as the ratio of the availability of fast food restaurants and convenience stores compared to grocery stores and produce vendors around respondents' homes. The researchers found that as the RFEI increased, so too did the prevalence of obesity. Other research from the United States supports the claim that access to convenience stores [12,13] and fast-food restaurants [13-16] is associated with obesity among adults while the presence of healthy food through grocery stores is a buffer to obesity [12,17,18].

Outside of the United States the evidence for such associations is limited and equivocal. For instance, the likelihood of being overweight/obese decreased among both girls and their fathers in Melbourne, Australia if they had a fast-food restaurant within 2 km of their home [19]. With each additional fast-food restaurant within 2 km of the home, the likelihood of being overweight/obese decreased by 14% for older girls. These findings are incongruent with the obesogenic environment model [10,20] and suggest that environmental associations with bodyweight status may vary by country or economic or political contexts. Canada provides an interesting comparison with the United States in this regard. Though the two countries are on the same continent, share many of the same values, and consume and enjoy many of the same products and foods, the rates of overweight and obesity differ. In 2004, 23% of Canadian adults were considered obese [21] while the prevalence of obesity was 32% in the United States [22]. Thus the question is whether food access and the density of fast-food restaurants is related to obesity rates in a Canadian sample.

Therefore, the purposes of this study were (a) to determine if the local food environment is associated with obesity in a Canadian context, and (b) if this association varies as a function of distance between food locations and people's homes. Using data from the Population Health Survey 2002 (PHS-2002) [23], we examined cross-sectional associations between the relative availability of different types of food retailers around individuals' homes and the prevalence of obesity among adults. We hypothesized that residents of areas with high fast-food access would be more likely to be obese than those in areas with relatively low access. Additionally, we hypothesized that these associations would be stronger for facilities that were more proximal (within 800 m) to the resident's home as opposed to more distal (within 1600 m).

## Methods

### Participants

The PHS-2002 was a telephone-administered survey conducted in the Capital Health region of Alberta, Canada between October and December 2002. At that time, the region included the City of Edmonton, the City of St. Albert, Strathcona County and Leduc County (including the City of Leduc and surrounding municipalities), with a total estimated population of 860,000. The target population included all individuals, 18 years or older, living in the Capital Health region. People who lived in homes without telephones and residents of institutions were excluded from this survey. A sample of 3,850 individuals was reached with a response rate of 59%. For the purposes of this study, only those individuals who lived within the City of Edmonton and who provided complete height and weight information were included (N = 2900). We certify that all applicable institutional and governmental regulations concerning the ethical use of human volunteers were followed during this research and institutional ethics board approval was obtained prior to data collection.

### Measures

#### Demographics

Since people living in a poor neighbourhood are much more likely to be exposed to fast-food outlets [24-28], less likely to have ready access to grocery stores [29,30] and more likely to be obese [31-33], then socioeconomic status (SES) is a potential covariate of any association between residential environments and obesity. Thus, measures of both individual- and neighbourhood-level SES were included in our analysis. Based on the respondent's postal code, data were extracted from the 2001 Census [34] and a neighbourhood SES index [35] was created by taking the sum of the z-scores of net educational level (the proportion of people with low education subtracted from the proportion of people with and high education aged 20 and over), median income of census families, and proportion of unemployed (unemployed people aged 15 and over as a percentage of people aged 15 and over who were in the labour force). Education was a stronger covariate of risk for obesity than household income in the PHS-2002 [23], therefore we used level of education attained by the respondent (less than high school, completed high school, some post secondary, completed college/technical school, completed university, completed post-bachelor university) as our sole indicator of individual-level SES.

#### Height and weight

Based on self-reported height and weight, the body mass index (BMI) was calculated and participants were classified as being obese if they had a BMI of 30 or greater [36].

#### Food Retailers

Information on location of food establishments in Edmonton for 2004 was supplied by the Health Inspection Division within Capital Health and/or found in the Alberta First Business Directory [37]. From these datasets, we selected supermarkets, fast food outlets, speciality food stores, and convenience stores according to the NAICS (North American Industry Classification System) codes. Supermarkets were defined as stores stocking fresh meat, wheat-based Western style bread, fruits, vegetables, and dairy milk, and had no required membership (free access). Fast food outlets were defined as restaurants with walk-up counter service selling predominantly pre-processed and prepared to order foods. Specialty food stores retailed miscellaneous specialty foods (e.g., ethnic, organic, or upscale/gourmet) not for immediate consumption. Convenience stores were defined as primarily engaged in retailing a limited line of goods that generally includes milk, bread, soda, and snacks. ArcGIS version 9.2 (ESRI Inc., Redlands, California, USA) was used to create buffers of 800 m and 1600 m around the points indicating the location of respondents' postal codes. To calculate the number of facilities (e.g., fast food restaurants, convenience stores, supermarkets) within each buffer, we used the Count Points in Polygon analysis tool [38]. A Retail Food Environment Index (RFEI) [11] was calculated for each respondent within both buffers. The RFEI was based on the following formula: *RFEI = (F+C)/G *where *F *represents the number of fast-food restaurants within a given radius; *C *represents the number of convenience stores (including convenience stores, gasoline stations with convenience stores and convenience neighbourhood stores that also sell selected grocery items) within a given radius; and *G *represents the number of grocery stores (including supermarkets, ethnic stores and upscale organic markets) within a given radius [11]. If no grocery store was found within a particular buffer, a constant of 1 was added to that case so that it remained in the analysis. A higher REFI would, therefore, indicate a more obesogenic food environment. Consistent with previous research [11], the RFEI for the 800 m buffer was then categorized as: below 3.0, 3.0 – 4.9, and 5.0 and above. For the 1600 m buffer, the categorization of RFEI was: below 6.0, 6.0 – 9.9, and 10.0 and above.

The average size of a residential neighbourhood in Edmonton is 1.07 km^2^, (the minimum area is 0.20 km^2^, the maximum area is 2.56 km^2^, with a Standard Deviation of 0.38 km^2^). Since we used buffers at radii of 800 m and 1600, they covered an area of 2.01 km^2 ^and 8.03 km^2 ^respectively. Therefore a buffer of 800 m covered more than the whole area of an average neighbourhood in the city (assuming the individual household was located in the centre of the neighbourhood), while capturing accessibility to grocery stores, fast food and convenience stores located within a resident's reach.

### Analysis

To examine the association of the RFEI with the prevalence of obesity, weighted logistic regression models were conducted for both the 800 m and 1600 m buffers. The models were weighted to reflect the age and sex distributions of the Capital Health region and adjusted for the respondent's age, sex, education level, and neighbourhood SES. Specifically, three models were computed for each buffer. Model 1 included the RFEI and no other covariates. Model 2 adjusted for neighbourhood SES while Model 3 included all covariates.

Before proceeding with our analysis, we determined whether scores may have been nested within neighbourhoods thus warranting a multilevel analysis. We first measured the degree of dependence by calculating the intra-class correlation between neighbourhood and BMI. A small intra-class correlation (ICC) was found, p = .02, suggesting that the BMI scores were independent of neighbourhood. We also considered whether we had sufficient cases within neighbbourhoods to support a multilevel analysis. Since the 2900 respondents represented 240 neighbourhoods across the City of Edmonton (*M *= 12.08 adults per neighbourhood), a multilevel analysis was not appropriate. With approximately 12 respondents per neighbourhood we were well below the recommended rules of thumb of 20 to 30 observations per group (neighbourhood) [39]. In fact 45% of the neighbourhoods had fewer than 10 respondents while 55 (23%) had 20 or more children. Therefore, the very small intra-class correlation combined with too few cases (observation) per neighbourhood (group), suggested we could proceed with our analysis without considering the potential effect of neighbourhoods in a multilevel model.

## Results

The median RFEI for adults in Edmonton was 4.00 within an 800 m buffer around their residence and 6.46 within a 1600 m buffer around their residence (see Table 1). The mean RFEI was 5.09 (SD = 6.31) within an 800 m buffer around the residence and 8.81 (8.77) within a 1600 m buffer around the residence. Approximately 14% of the respondents were classified as being obese. This rate varied by RFEI classification with a lower proportion of people being classified as obese in the lowest RFEI category (12.7%, 13.7%) relative to the highest category (16.3%, 16.1%) for both the 800 m and 1600 m buffers respectively.

**Table 1:** Obesity Prevalence by Retail Food Environment Index for 800 m and 1600 m Buffers

Buffer		% of Residents	% Obese	Mean (SD) RFEI	Median RFEI
800 m				5.09 (6.31)	4.00
	RFEI below 3.0	38.3	12.7		
	RFEI 3.0–4.9	21.8	14.6		
	RFEI 5.0 and above	38.9	16.3		
1600 m				8.81 (8.77)	6.46
	RFEI below 6.0	41.6	13.7		
	RFEI 6.0–9.9	31.4	14.2		
	RFEI 10 and above	27.0	16.1		

RFEI = Retail Food Environment Index.

RFEI within 800 m of the home was negatively associated with obesity prevalence (see Table 2). Specifically, the odds of a resident being obese were significantly lower (OR = 0.75, 95%CI 0.59 – 0.95) if they lived in an area with the lowest RFEI (below 3.0) in comparison to the highest RFEI (5.0 and above). These associations existed regardless of the covariates included in the model. Based upon the Nagelkerke R-square statistic, the complete model accounted for approximately 4% of the variance in obesity. No significant associations were observed between RFEI within a 1600 m buffer of the home and obesity.

**Table 2:** Association Between Obesity Prevalence and Retail Food Environment Index

	Model 1	(*n *= 2897)	Model 2	(*n *= 2897)	Model 3	(*n *= 2890)
	OR	95% CI	OR	95% CI	OR	95% CI
	800 m Buffer					

RFEI below 3.0	0.75	0.59–0.95	0.75	0.59–0.95	0.74	0.59–0.94
RFEI 3.0–4.90	0.87	0.67–1.15	0.86	0.65–1.13	0.81	0.61–1.06
RFEI 5.0 and above	1.00		1.00		1.00	

	1600 m Buffer					

RFEI below 3.0	0.83	0.64–1.06	0.83	0.65–1.07	0.85	0.66–1.10
RFEI 3.0–4.90	0.86	0.66–1.13	0.89	0.68–1.16	0.93	0.71–1.22
RFEI 5.0 and above	1.00		1.00		1.00	

*Note*. Model 1 includes no covariates. Model 2 includes neighbourhood SES index. Model 3 includes neighbourhood SES index, age, sex, and education level. RFEI = Retail Food Environment Index.

## Discussion

In this study we explored whether the relative availability of different types of food retailers around people's homes was associated with obesity among adults in Edmonton, Canada, and if this association varied as a function of distance between food locations and people's homes. We found that the lower the ratio of fast-food restaurants and convenience stores to grocery stores and produce vendors near the home, the lower the odds of being obese. This association existed for establishments within an 800 m buffer around people's homes but not for establishments within 1600 m of their homes. Thus the proximity of the obesogenic environment [10] to individuals appears to be an important factor in their risk for obesity. Such findings may help explain the observation that geographic concentration of fast-food restaurants is associated with mortality and hospital admissions for acute coronary events among Canadians living in the Province of Ontario [40].

Our finding that the local food environment is associated with risk for obesity is consistent with previous research in the United States [12-18,41]. In particular, our study and findings were very similar to one conducted in California [11] where the average RFEI was 4.5 and a 20 percent difference in prevalence of obesity was observed between the lowest and highest RFEI groups within a buffer of approximately 800 m for urban residents and 1600 m for suburban residents. For instance, we found that an Edmonton resident is exposed to approximately 4 (median) to 5 (mean) times more fast-food restaurants and convenience stores within 800 m of their home than grocery stores and produce vendors. Within the 800 m buffer, the difference in prevalence of obesity was approximately 28% (i.e., 12.7% vs. 16.3%). One important distinction between the two studies is how the problem of missing data was addressed. Specifically, if no grocery stores or produce vendors were present within the buffer, the California researchers opted to exclude these cases from the RFEI calculation but yet include them in the overall frequency estimates. We addressed this issue by adding a constant of 1 to the grocery store/produce vendor variable for such cases so that we could calculate an RFEI for all cases. We did this so that any participant who was exposed to a fast-food restaurant was included in our analysis regardless of whether grocery stores were present in their immediate vicinity or not. This procedure resulted in a greater proportion of residents in our study being in the lowest and highest RFEI categories in comparison to the California study. For instance we had 38% and 39% of participants in the lowest and highest categories while in California the proportions were 25% and 26% respectively and 28% missing cases.

Risk for obesity is associated with urban form [42-44] and SES [31-33]. When neighbourhood SES was entered as a covariate in our analysis, we saw no attenuation of the association between the relative food index and obesity within the 800 m buffer. A slight attenuation may have occurred with the inclusion of household education level in the third model, but the association still remained. Thus, the obesogenic effect of access to unhealthy food appears to be independent of SES. These findings suggest that improving the retail food environment, regardless of neighbourhood- and individual-level SES, could be an effective strategy for decreasing the prevalence of obesity among adults.

Strengths of the study include the large representative sample and the use of objective criteria for defining the food environment. However, this study is not without limitations, including the cross-sectional design and use of self-reported height and weight. For instance, people often under-report height and over-report weight [45]. Though the prevalence of obesity observed in our study (14%) was similar to that (15.2%) found for Canadians through self-reports in 2002 [46], both rates are much lower than the 23% recorded with actual measures in 2004 [21]. It is unlikely, however, that this self-report bias would vary as a function of the food environment (independent of SES) in which people live and thus should not have influenced our findings. On a related note, our contextual SES variable was modeled at the neighbourhood level instead of at the level of the buffers. For respondents located close to the boundary between two neighbourhoods with different aggregated SES, this procedure may obscure a potential role of SES in attenuating the association between the RFEI and obesity. Finally, we have no information on the fast-food consumption habits of the participants so it is impossible to determine if they were actually consuming such foods and how they were traveling to the establishments. Since approximately 70% of fast-food purchases in the United States are done through takeout [47], it is possible that part of the association between relative fast-food access and obesity in this study is related to the mode of transportation (car as opposed to walking) involved in the visit to the restaurant. That is, people are being sedentary while they are "hunting and gathering" their caloric-dense food. Future research is needed to investigate associations between food environment, food retail usage, and food consumption patterns.

## Conclusion

In summary, the current study was the first to find that relative availability of different types of food retailers around peoples' homes was associated with obesity in a Canadian context. Similarly, it was among the first to document such associations outside of the United States. As our findings held regardless of neighbourhood- and individual-level SES, a plausible policy option for decreasing the prevalence of obesity among adults is improving the retail food environment, possibly through zoning by-laws. A recent systematic review of urban environment and healthy weights [48] revealed a variety of policy options have been proposed in Canada, but not systematically implemented or evaluated. Thus, future research on effectiveness of interventions to improve health and weight outcomes at the urban environment level is warranted.

## Competing interests

The authors declare that they have no competing interests.

## Authors' contributions

All authors were involved in various stages of study design. NC conducted the spatial analysis and oversaw the database. JCS conducted the statistical analysis and wrote the paper. All authors commented on drafts and approved the final text.

## Pre-publication history

The pre-publication history for this paper can be accessed here:


